# A Genomic-Clinicopathologic Nomogram Predicts Survival for Patients with Laryngeal Squamous Cell Carcinoma

**DOI:** 10.1155/2019/5980567

**Published:** 2019-11-20

**Authors:** Jie Cui, Qingquan Wen, Xiaojun Tan, Zhen Chen, Genglong Liu

**Affiliations:** ^1^Department of Head and Neck Surgery, Affiliated Cancer Hospital & Institute of Guangzhou Medical University, Guangzhou 510095, Guangdong Province, China; ^2^Department of Pathology, Affiliated Cancer Hospital & Institute of Guangzhou Medical University, Guangzhou 510095, Guangdong Province, China; ^3^Department of Intensive Care Unit, Shunde Hospital, Southern Medical University (The First People's Hospital of Shunde), Foshan 528308, Guangdong Province, China

## Abstract

**Background:**

Long noncoding RNAs (lncRNAs), which have little or no ability to encode proteins, have attracted special attention due to their potential role in cancer disease. We aimed to establish a lncRNA signature and a nomogram incorporating the genomic and clinicopathologic factors to improve the accuracy of survival prediction for laryngeal squamous cell carcinoma (LSCC).

**Methods:**

A LSCC RNA-sequencing (RNA-seq) dataset and the matched clinicopathologic information were downloaded from The Cancer Genome Atlas (TCGA). Using univariable Cox regression and least absolute shrinkage and selection operator (LASSO) analysis, we developed a thirteen-lncRNA signature related to prognosis. On the basis of multivariable Cox regression analysis results, a nomogram integrating the genomic and clinicopathologic predictors was built. The predictive accuracy and discriminative ability of the inclusive nomogram were confirmed by calibration curve and a concordance index (C-index), and compared with the TNM staging system by C-index and receiver operating characteristic (ROC) analysis. Decision curve analysis (DCA) was conducted to evaluate the clinical value of our nomogram.

**Results:**

Thirteen overall survival- (OS-) related lncRNAs were identified, and the signature consisting of the selected thirteen lncRNAs could effectively divide patients into high-risk and low-risk subgroups, with area under curves (AUC) of 0.89 (3-year OS) and 0.885 (5-year OS). Independent factors derived from multivariable analysis to predict survival were margin status, tumor status, and lncRNA signature, which were all assembled into the nomogram. The calibration curve for the survival probability showed that the predictions based on the nomogram coincided well with actual observations. The C-index of the nomogram was 0.82 (0.77-0.87), and the area under curve (AUC) of the nomogram in predicting overall survival (OS) was 0.938, both of which were significantly higher than the traditional TNM stage. Decision curve analysis further demonstrated that our nomogram had larger net benefit than TNM stage.

**Conclusion:**

An inclusive nomogram for patients with LSCC, comprising genomic and clinicopathologic variables, generates more accurate estimations of the survival probability when compared with TNM stage alone, but more data are needed before the nomogram is used in clinical practice.

## 1. Introduction

As an aggressive malignancy, laryngeal squamous cell carcinoma (LSCC) accounts for 85–95% of all laryngeal cancer and is one of the most prevalent cancers in the head and neck region [1]. According to age-adjusted morbidity estimates in the United States, the incidence of LSCC is 5.4 per 100,000 male residents and 1.1 per 100,000 female residents [2]. In Europe, these figures slightly shift; it is higher for men with 8.8 incidents per 100,000 inhabitants and lower for women with 0.8 incidents per 100,000 inhabitants, respectively [3]. It has been reported that the 5-year overall survival rate is approximately 60% for treated laryngeal cancer patients [4]. Although multimodal therapies have been applied in LSCC, the survival outcome is still unsatisfactory, especially for advanced laryngeal carcinoma [5].

Currently, the existing 8th edition of the American Joint Committee on Cancer (AJCC) tumor-node-metastasis (TNM) staging system [6] based on anatomical information is often adopted when talking about the estimated prognosis of patients. LSCC consists of a heterogeneous histological subtype with extensive clinical course variations [7]. As a result, a significant proportion of patients with inaccurate staging may receive inadequate treatment or overtreatment. In particular, understaging might lead to recurrence or even death after surgery; conversely, overstaging is likely to subject a patient to needless adjuvant chemoradiotherapy. Hence, identifying reliable and novel markers/models to improve the accuracy of prediction in LSCC patients is very urgent and is necessary for optimizing treatment planning for the benefit of patients.

As previous genome researches revealed, more than ninety percent of the human genome is actively transcribed into noncoding RNAs (ncRNAs) [8]. Conventionally, this ncRNA family is loosely classified into two groups based on molecular size: small ncRNA (e.g., microRNA; with a length of less than 200 nt) and long noncoding RNA (lncRNA; with a length of more than 200 nt) [9]. Unlike protein-coding RNAs, the expression patterns of the lncRNAs are more specific. A large number of researches have reported the diverse biological functions of lncRNAs, such as tumorigenesis and tumor progression, as well as metastasis [10]. lncRNA can be considered as a new cancer biomarker, which represents a large number of potential molecular drivers in human cancer disease [11]. In the past few years, lncRNA signatures have been reported to evaluate the prognosis of cancers, including head and neck cancer, cervical carcinoma, and gastric cancer [12–14]. However, the lncRNA signature that can predict the overall survival (OS) outcome of LSCC has not been found yet.

In the current study, we hypothesized that an inclusive nomogram containing genomic and clinicopathological factors can improve the accuracy of survival prediction. By mining the expression data of lncRNAs in The Cancer Genome Atlas (TCGA), we identified lncRNAs that were significantly related to survival outcomes and then developed a lncRNA signature. An inclusive nomogram for predicting survival status was established by further integrating the lncRNA signature with clinicopathological factors. We assessed the predictive ability and clinical application of the nomogram and compared it to the TNM stage. In addition, we evaluated the prediction effect of the nomogram in clinical subgroups (advanced LSCC and early LSCC).

## 2. Materials and Methods

### 2.1. Collection of Publicly Available Data from TCGA

A LSCC RNA-sequencing (RNA-seq) dataset and relevant clinicopathological information including the age, sex, smoking history, alcohol history, number of lymph nodes (LN), number of positive LNs, lymph node ratio (LNR), margin status, tumor status, histologic grade, T stage, N status, TNM stage, mutation count, fraction genome altered, and overall survival (OS) time were downloaded from a publicly available TCGA database (https://gdc.cancer.gov/). A total of 109 patients with complete follow-up data were extracted, which was recorded before April 14, 2019. The clinical end point was overall survival (OS), defined as the time from surgery to death. In addition, patients who were alive at the last follow-up are considered as censored observations.

Given that the expression level of lncRNAs is relatively low compared with noncoding RNA, it is likely that some lncRNAs have not been analyzed during the sequencing procedure of lncRNAs. Considering this possibility, we defined lncRNAs as being expressed abundantly when its expression level is above 0 and occurs more than 50% in the total samples. The final expression level of lncRNAs was represented as log_2_(*x* + 1) of the original expression level.

### 2.2. Construction and Confirmation of a lncRNA Signature

First, a moderated *t*-statistics method and the Benjamini-Hochberg procedure are used to identify distinct differential lncRNAs between normal tissues and LSCC tissues, with *P* < 0.05 and the false discovery rate (FDR) < 0.05 for filtration. Next, univariable Cox regression analysis is used to select prognostic-related lncRNAs with a statistical significance of *P* < 0.01. After primary filtering, a Least Absolute Shrinkage and Selector Operation (LASSO) analysis is established to select candidate lncRNAs with a penalty parameter tuning adjusted by 10 times cross validation [15], then a signature based on these well-selected lncRNAs is developed.

The risk score formula is generated by integrating these prognostic-related lncRNA, weighted by their respective LASSO regression coefficients. According to this formula, each patient's risk score was calculated, and patients were divided into high-risk or low-risk group on the basis of the optimal cutoff point, which was adopted in the maximum sensitivity and specificity by using a (time-independent) receiver operating characteristic (ROC) curve. The survival differences between the high-risk group and low-risk group are further compared by the Kaplan-Meier analysis with a log-rank test. Stratified analysis based on various clinical characteristics is conducted to evaluate the discrimination ability of the lncRNA signature.

### 2.3. Function Prediction of the Prognostic lncRNAs

In TCGA dataset, according to their expression level, the Pearson correlation algorithm is performed between the identified lncRNAs and the differential protein-coding genes (mRNAs) (a moderated *t*-statistics method and the Benjamini-Hochberg procedure are used to identify distinct differential mRNAs between normal tissues and LSCC tissues, with *P* < 0.05 and the false discovery rate (FDR) < 0.05 for filtration). The correlation coefficient > 0.4 (*P* < 0.001) is considered significant correlation. The potential biological processes of the lncRNA target genes are investigated by using Gene Ontology (GO) and Kyoto Encyclopedia of Genes and Genomes (KEGG). DAVID is a common bioinformatics tool (http://david.abcc.ncifcrf.gov/, version 6.8) [16], which is used to explore the biological functions of the selected lncRNAs. *P* values corrected with a false discovery rate (FDR) < 0.05 for GO analysis and KEGG pathways are considered remarkably enriched functional annotations.

### 2.4. Genomic-Clinicopathologic Nomogram

To build a genomic-clinicopathologic nomogram, we used univariate and multivariate Cox regression analysis to identify clinical risk parameters associated with survival. Then, the lncRNA-based signature, together with the risk parameters, were used to develop a genomic-clinicopathologic nomogram.

The performance of the model was assessed by calibration and discrimination via a bootstrap method with 1000-iteration resampling. Discrimination is the model's ability to differentiate between patients who survived versus those who did not. The concordance index (C-index) was calculated to evaluate the discrimination. Besides, we illustrated the discrimination by dividing the dataset into three groups according to the scores generated by the nomogram. We plotted a Kaplan-Meier curve for all three groups. In addition, calibration curves with a plot of the observed outcomes against the nomogram-predicted probabilities were graphically evaluated.

Furthermore, we used ROC analysis to investigate and compare the discrimination ability of the nomogram with TNM stage or lncRNA signature. Decision curve analysis (DCA) was used to evaluate the clinical usefulness and net benefit of the predictive model, and compared with traditional TNM staging or lncRNA signature [17]. Finally, we assessed the predictive accuracy of the comprehensive nomogram in clinical subgroups (advanced LSCC and early LSCC).

### 2.5. Statistical Analysis

Categorical variables are provided as proportions (%). Continuous variables are described as medians (interquartile ranges (IQRs)) if the distribution was nonnormal and as means (standard deviations (SDs)) if the distribution was normal.

If there were missed values in some of the potential predictors, these missing data would be imputed, as complete case analysis would improve the statistical power and reduce a potentially biassed result [18]. Multiple imputation (MI) was used to interpolate the missing data as the missing data were considered missing at random after analyzing patterns of them [19]. We used the Markov Chain Monte Carlo (MCMC) function to perform MI, and we selected five iterations to account for possible simulation errors.

The LASSO algorithm was conducted with “glmnet” packages, and ROC analysis was done with “timeROC” and “survivalROC” packages. The nomogram and calibration plots were generated with “rms” packages, and DCA was performed with the “stdca.R”.

SPSS statistics 22.0 and R software (R version 3.5.2) were used to conduct the statistical analysis. A two-sided *P* < 0.05 would be recognized as statistically significant.

## 3. Results

### 3.1. Demographic Parameters and OS Outcome of LSCC Patients

In the current study, 109 LSCC patients with available lncRNA data and clinicopathological characteristics were included. The basic clinicopathological features of these LSCC patients were summarized in Table 1. The median OS time was 23.32 months (from 2.53 to 210.81 months). Of all the 109 LSCC patients, 49 patients (45%) died during follow-up and the 3-year and 5-year OS rates were 79.7% (71.7–87.7%) and 64.5% (54.7–74.3%), respectively.

### 3.2. Construction and Confirmation of a lncRNA Signature

**Figure 1:**
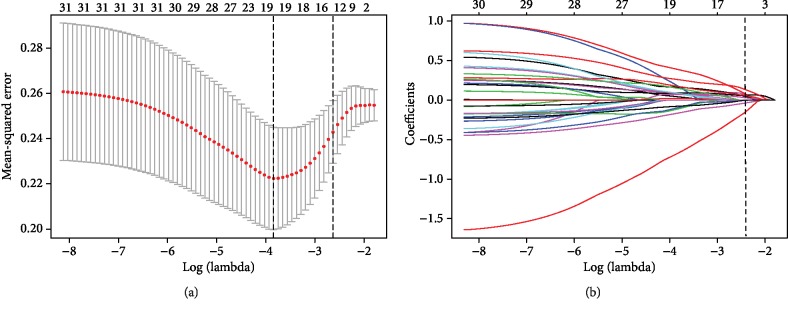
Thirteen lncRNAs selected by LASSO Cox regression analysis. (a) The two dotted vertical lines are drawn at the optimal values by the minimum criteria (left) and 1 − s.e. criteria (right). Details are provided in Materials and Methods. (b) LASSO coefficient profiles of the 31 lncRNAs. A vertical line is drawn at the optimal value by 1 − s.e. criteria and results in thirteen nonzero coefficients. Thirteen lncRNAs—AC007907.1, AC025419.1, AC078993.1, AC090241.2, AL158166.1, AL355974.2, AL596330.1, HOXB-AS4, KLHL6-AS1, LHX1-DT, LINC00528, LINC01436, and TTTY14—with coefficients 0.2102, 0.0045, 0.1377, -0.3675, -0.0652, 0.0180, 0.1208, 0.0969, 0.2227, 0.1541, -0.0647, -0.0750, and -0.1360, respectively, were selected in the LASSO Cox regression model.

Based on the primary filter criteria mentioned in Materials and Methods, we obtained a list of 1160 different lncRNAs (Supplementary [Supplementary-material supplementary-material-1]). Then, using univariable Cox regression analysis, we identified 31 prognostic-related lncRNAs (Supplementary [Supplementary-material supplementary-material-1]). Finally, the LASSO algorithm was used to shrink and pick out the OS-related lncRNAs (Figure 1), which were AC007907.1, AC025419.1, AC078993.1, AC090241.2, AL158166.1, AL355974.2, AL596330.1, HOXB-AS4, KLHL6-AS1, LHX1-DT, LINC00528, LINC01436, and TTTY14, to build a lncRNA-based signature.

**Figure 2:**
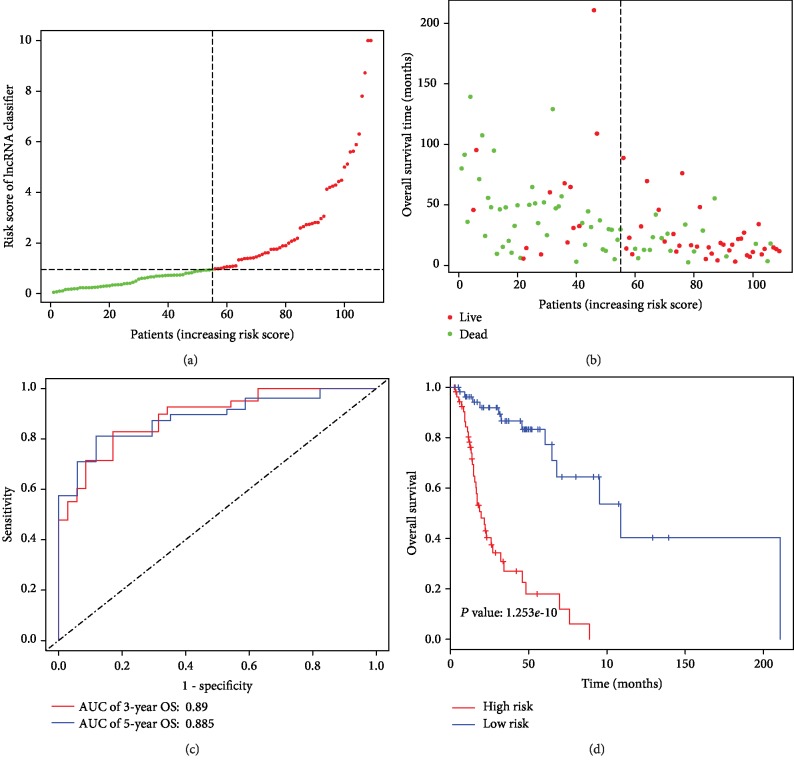
Development of lncRNA signature for the prediction of survival in LSCC patients. (a and b) Distribution of lncRNA-based classifier risk score. (c) Time-independent ROC curves with AUC values to evaluate predictive efficacy of the lncRNA signature risk score. (d) The Kaplan-Meier estimates of the patients' survival status and time using the optimal lncRNA signature risk score cutoff which divided patients into low-risk and high-risk groups.

In order to better investigate the value of the lncRNA signature in predicting prognosis, a risk score was established, with the coefficients weighted by the LASSO Cox regression model. The risk score was generated as follows: risk score = (0.2102 expression level of AC007907.1) + (0.0045 expression level of AC025419.1) + (0.1377 expression level of AC078993.1) + (−0.3675 expression level of AC090241.2) + (−0.0652 expression level of AL158166.1) + (0.0180 expression level of AL355974.2) + (0.1208 expression level of AL596330.1) + (0.0969 expression level of HOXB ‐ AS4) + (0.2227 expression level of KLHL6 ‐ AS1) + (0.1541 expression level of LHX1 ‐ DT) + (−0.0647 expression level of LHX1 ‐ DT) + (−0.0750 expression level of LINC01436) + (−0.1360 expression level of TTTY14). Using ROC curve to generate the optimal cutoff value for the risk score, patients were divided into the high-risk group and low-risk group. As was shown at Figure 2, patients with the high-risk score were more likely to die and had shorter OS time than patients with a low-risk score (19.74 vs. 108.9 months, HR = 5.79, 95% CI: 3.18-10.54, *P* < 0.0001). The lncRNA signature had a superior prediction effect, with an AUC of 0.89 for 3-year OS and an AUC of 0.885 for 5-year OS (Figure 2(c)). Additionally, a 13-lncRNA signature in subsets of patients with different clinical variables were analyzed by stratification analysis. When stratified according to clinical variables (tumor size, node status, and TNM stage), a 13-lncRNA signature remained a clinically and statistically significant prognostic model (Supplementary [Supplementary-material supplementary-material-1]).

### 3.3. Functional Enrichment Analysis of the 13 lncRNAs

**Figure 3:**
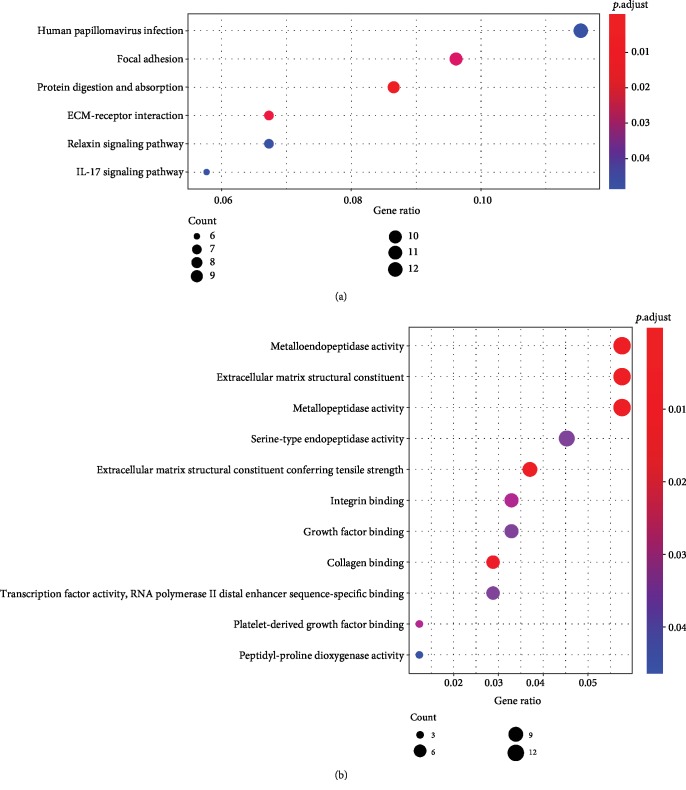
Functional annotation of the prognostic lncRNAs. Significantly enriched using the coexpressed mRNAs of the lncRNAs in GO analysis (a) and KEGG pathway analysis (b).

To investigate the potential function of the 13 lncRNAs, a total of 237 protein-coding genes (mRNAs) were significantly correlated with at least one of the 13 lncRNAs (Pearson coefficient > 0.4, *P* < 0.001), which was considered eligible for pathway enrichment. The 13 lncRNAs were mainly related with human papillomavirus infection, focal adhesion, and protein digestion and absorption (Figure 3(a)). And KEGG pathway analysis revealed that 13 lncRNA-related target genes were mainly enriched in metalloendopeptidase activity, extracellular matrix structural constituents, and metallopeptidase activity (Figure 3(b)).

### 3.4. Development of a Genomic-Clinicopathologic Nomogram Predicting OS in LSCC Patients

**Figure 4:**
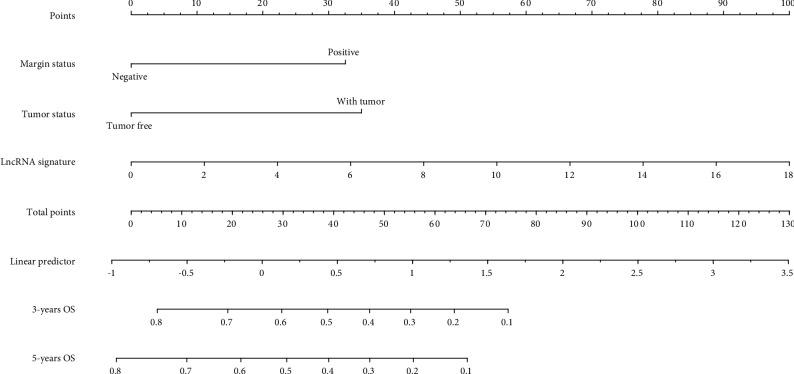
Nomogram for predicting 3-year and 5-year survival probability of LSCC after laryngectomy. To estimate risk, calculate points for each variable by drawing a straight line from the patient's variable value to the axis labeled “Points.” Sum all points and draw a straight line from the total point axis to the 3-year and 5-year survival axis.

Using univariate Cox analysis, we identified that four variables, including sex, margin status, tumor status, and lncRNA signature, were associated with survival probability (Table 2). Multivariable analysis continued to verify that margin status, tumor status, and lncRNA signature, were independent risk factors for OS. Based on multivariate analysis of OS, a genomic-clinicopathological nomogram was built to predict OS in 3 and 5 years (Figure 4). The C-index of the nomogram for OS prediction was 0.82 (0.77-0.87) (Table 3). The calibration plot of OS probabilities for 3 and 5 years showed the best consistency between the nomogram prediction and the actual observations (Supplementary [Supplementary-material supplementary-material-1]). Additionally, the Kaplan-Meier curve was used to analyze the discrimination ability of the nomogram to predict OS, and a significant statistical difference was found among the three groups (log-rank *P* < 0.0001) (Supplementary [Supplementary-material supplementary-material-1]).

### 3.5. Comparison of Predictive Performance and Clinical Usefulness between Nomogram and TNM Staging or lncRNA Signature

**Figure 5:**
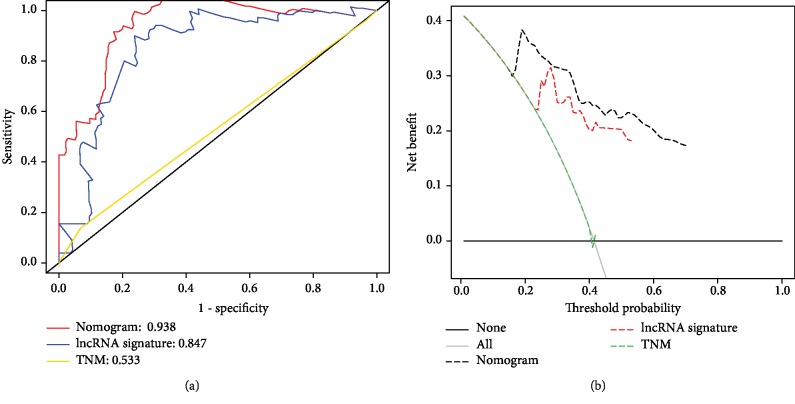
ROC curves compare the prognostic accuracy of the nomogram with TNM staging or lncRNA signature in predicting survival probability (a). Decision curve analysis for the nomogram, TNM staging, and lncRNA signature in prediction of prognosis of patients (b).

To evaluate the predictive ability of the nomogram, we compared the nomogram model with the AJCC TNM staging model and lncRNA signature. As was shown at Table 3, the C-index of the nomogram was higher than that of TNM staging (0.53 (0.45-0.61)) and lncRNA signature (0.78 (0.71-0.85)). Likelihood ratio test, linear trend *χ*^2^ test, and Akaike information criterion all showed that the nomogram was better than TNM staging or lncRNA signature. ROC analysis also indicated that the nomogram (AUC 0.938) had a higher prediction efficiency than TNM staging (AUC 0.533) or lncRNA signature (AUC 0.847) (Figure 5(a)). Finally, DCA was used to compare the clinical usability of the nomogram to that of traditional TNM staging and lncRNA signature. According to the continuity of potential death threshold (*x*-axis) and the net benefit of risk stratification using the model (*y*-axis), DCA graphically revealed that the nomogram was superior to the traditional TNM staging or lncRNA signature (Figure 5(b)).

**Figure 6:**
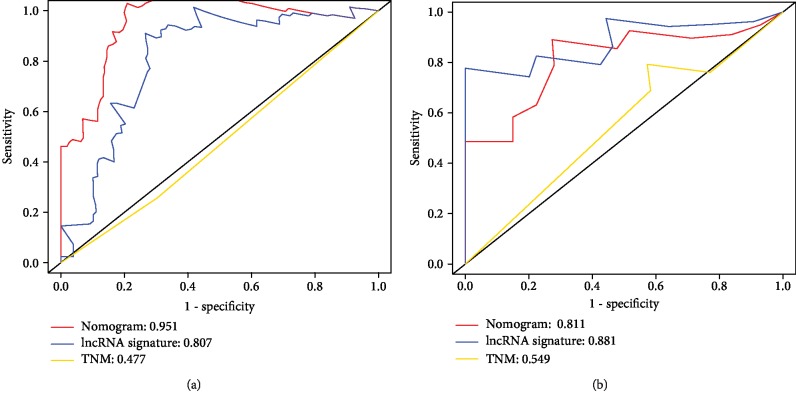
ROC curve analyses for survival prediction in subgroups of patients with LSCC. (a) Advanced LSCC subgroup and (b) early LSCC subgroup.

**Figure 7:**
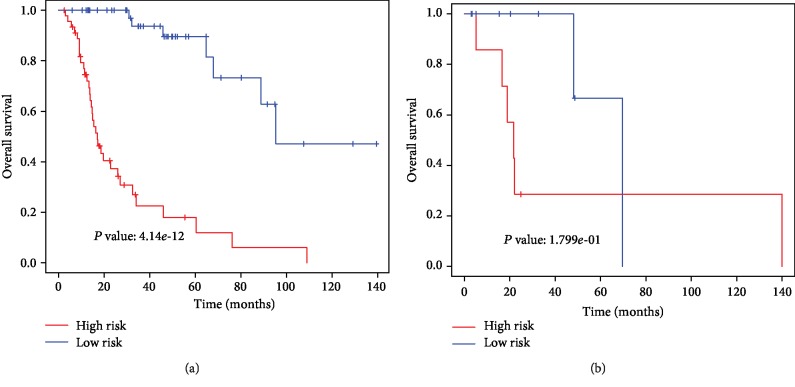
The Kaplan-Meier estimates of patients' survival status and time using the optimal nomogram risk score cutoff which divided patients into low-risk and high-risk groups in subgroups of patients with LSCC. (a) Advanced LSCC subgroup and (b) early LSCC subgroup.

Furthermore, ROC analysis in clinical subgroups (advanced LSCC and early LSCC) was conducted to evaluate the discrimination ability of the nomogram. As shown in Figure 6, encouragingly, the nomogram presented good discrimination ability in the advanced LSCC subgroup (AUC 0.951; Figure 6(a)) and the early LSCC subgroup (AUC 0.811; Figure 6(b)). Moreover, the patients in each subgroup were classified into a low-risk group and a high-risk group by the best cutoff values. Notably, the low-risk group was more likely to survive between the two subgroups (Figures 7(a) and 7(b)).

## 4. Discussion

Analyzing the LSCC RNA-sequencing (RNA-seq) dataset and relevant clinical parameters of 109 LSCC patients from TCGA, we identified thirteen lncRNAs related to OS. On the basis of these lncRNAs, we developed a lncRNA signature, which could accurately categorized patients into high-risk status and low-risk status. Additionally, we built a visually inclusive nomogram, integrating lncRNA signature and clinicopathologic variables to predict survival in LSCC patients who underwent surgery resection. The nomogram effectively predicted survival rate, with a bootstrapped corrected C-index of 0.73 and an AUC of 0.938, which possessed better predictive ability and clinical usability than TNM stage alone.

Increasing the number of studies have found that lncRNAs may be exploited as potential effective biomarkers in diagnosis, progression, and prognosis of LSCC [20–22]. Basing on a comprehensive lncRNA profile for LSCC, Shen et al. [20] identified AC026166.2-001 and RP11-169D4.1-001 as new lncRNAs with an accurate diagnosis value for LSCC that were independent factors for prognosis and may be potential therapeutic targets. A study of a lncRNA microarray by Chen et al. [21] found that lncRNA AC008440.10 was significantly correlated with LSCC stage, lymph node metastasis (LNM), and survival time. Recently, Zhao et al. [22] confirmed that LINC00668 was upregulated in LSCC and probably aggravated the malignant phenotypes of cells in vitro, and the authors speculated that LINC00668 may enhance the stability of RAB3B mRNA by binding its 3′UTR. Notably, Feng et al. [23] collected data from the open Gene Expression Omnibus (GEO), and they reported that 18-mRNA and one-lncRNA modules were associated with disease-free survival (DFS) of LSCC patients and it effectively divided patients into a high-risk group and a low-risk group with different DFS outcomes, independent of patient age and tumor grade. Similarly, Bai et al. [24], using data from GEO, constructed a potential panel out of two lncRNA signatures, including RP11-169K16.4 and RP11-107E5.3, to predict the recurrence of patients with laryngeal cancer and confirmed that it was an independent predictor of laryngeal cancer patients. These studies suggested the potential clinical implications of lncRNA in improving the prognosis prediction of LSCC. However, it should be noted that the lncRNA signature predicting the overall survival (OS) outcome of LSCC has not been reported yet. Hence, in the current study, using the TCGA database containing large-scale lncRNA expression data, we aimed to identify OS-related lncRNAs and establish a lncRNA signature, which may be more valuable for LSCC patients to optimize tailored treatment in the era of precision medicine.

To our knowledge, this is the first study that has constructed an inclusive nomogram, combining lncRNA signature and clinicopathologic factors, for predicting survival probability in patients with LSCC. We built a lncRNA signature consisting of AC007907.1, AC025419.1, AC078993.1, AC090241.2, AL158166.1, AL355974.2, AL596330.1, HOXB-AS4, KLHL6-AS1, LHX1-DT, LINC00528, LINC01436, and TTTY14 that could effectively classify patients into a high-risk group with a shorter OS and a low-risk group with a longer OS. Using stratified analysis, the lncRNA signature has shown a perfect discrimination ability regardless of tumor size, node status, and TNM stage. Additionally, we identified three independent predictors, namely, margin status, tumor status, and lncRNA signature, which were all embedded into the nomogram. In this study, in consideration of homogeneity and the ability of discrimination and risk stratification of the model, the performance of the nomogram in predicting survival probability is superior to the TNM staging system. The advantage of the current nomogram is that it integrated genomic and clinicopathological variables, which are important for predicting survival risk but cannot be obtained by the TNM staging system. Remarkably, DCA results showed that a LSCC survival-related treatment decision based on the nomogram led to more net benefits than the treatment decision based on TNM staging or lncRNA signature, or treating either all patients or none. Taken together, the present nomogram would be clinically useful for the clinicians in tailoring a survival-associated treatment decision.

It is also worth mentioning that an important feature of our inclusive nomogram may be the ability to stratify clinical subgroups, including early LSCC and advanced LSCC. Patients diagnosed with early LSCC are generally considered to have a low survival risk, and therefore do not receive adjuvant treatment after radical resection.

However, some patients in the clinically low-risk subgroup (early LSCC) are at high risk of survival, and they are likely to benefit from adjuvant treatment or an intensive follow-up plan. Likewise, patients diagnosed with advanced LSCC are usually identified to have a high-risk survival status and need to receive adjuvant therapy after undergoing laryngectomy. Nevertheless, several patients in the clinically high-risk subgroup (advanced LSCC) are at low risk of survival, and they may not benefit from adjuvant therapy or an intensive follow-up plan. It is an arduous challenge to accurately predict the survival risk of patients. Encouragingly, our inclusive nomogram presented a good discrimination ability in early LSCC and advanced LSCC subgroups. Hence, our nomogram will probably benefit a large proportion of the patients who might be considered at high risk of survival in the early LSCC subgroup or might be considered at low risk of survival in the advanced LSCC subgroup.

Among the thirteen OS-related lncRNAs, LINC01436 and TTTY14 have been previously reported to be related with cancers, such as in non-small-cell lung cancer (NSCLC), oral squamous cell carcinoma (OSCC), and gastric cancer [25–27]. LINC01436 has been reported to be a potential prognostic biomarker for NSCLC patients [25]. In terms of mechanisms, LINC01436, acting as a microRNA- (miR-) 30a-3p sponge, regulated the expression level of its target gene EPAS1. TTTY14 (testis-specific transcript, Y-linked 14) was thought to be associated with human papillomavirus- (HPV-) induced tumorigenesis; the expression level of TTTY14 was observably different between an HPV active group and an HPV inactive/negative group [28], in accordance with our functional enrichment analysis. In addition, TTTY14 was identified as significantly correlated with overall survival in OSCC and gastric cancer [26, 27]. Hence, further characterization of molecules such as AC007907.1, AC025419.1, AC078993.1, AC090241.2, AL158166.1, AL355974.2, AL596330.1, HOXB-AS4, KLHL6-AS1, LHX1-DT, LINC00528, LINC01436, and TTTY14 will provide a new perspective for the development and progress of LSCC and will aid in finding potential therapeutic targets for LSCC patients.

Consistent with previous studies, margin status was found to have a significant association with survival among patients with LSCC in the present study [29, 30]. Published trials about LSCC did not report that tumor status was an independent risk factor for OS. However, it was related to the prognosis of LSCC in our study. Tumor status has been confirmed that it was an independent prognostic factor for survival in hepatocellular carcinoma (HCC) [31]. Additionally, we identified that being male was positively associated with OS probability in the univariate Cox analysis, inconsistent with previous trials that being male was a poor prognosis for LSCC [32, 33]. Nevertheless, the effect of gender on the prognosis of OS was not statistically significant in multivariable Cox analysis. In addition to these clinical factors, as expected, the lncRNA signature was an effective independent prognostic factor for the prediction of patients with LSCC.

Although our nomogram demonstrated impressive performance in LSCC survival prediction, there are specific limitations associated with our trial. First, the presented nomogram was based only on TCGA database with limited simple sizes for LSCC, and is not yet suitable for general use prior to validation of the predictive models with external datasets. So, external and multicenter prospective cohorts with large sample sizes are still needed to validate the clinical application of our model.

Second, missing variables were a source of defect in this evaluation. This included extracapsular spread [34, 35], lymphovascular invasion status [35], perineural invasions [35], and human papillomavirus (HPV) [36, 37] as important prognostic parameters for LSCC patients, which were not well recorded in the TCGA database. Notably, our functional enrichment analysis found that the prognostic lncRNAs were significantly associated with human papillomavirus. Published researches and meta-analyses indicated that HPV-positive laryngeal cancer patients are sensitive to radiotherapy and chemotherapy and showed inferior survival [36, 37]. Hence, we recommend that future studies should add value to those factors in a multivariable prediction model to improve the accuracy of prediction in LSCC patients.

Third, our choice of factors was limited to those available in our database. On account of the anonymous database, we cannot extend our database with variables such as race, insurance status, comorbidity, hemoglobin level, albumin, tumor hypoxia, and TP53 mutation, which were frequently reported prognostic factors of patients with LSCC [38–40]. Further efforts to incorporate more patient-specific and tumor-specific molecular factors will potentially help to improve the performance of the present model.

Fourth, we do not explore the underlying biological function and pathways of the prognostic lncRNAs, so further studies are needed to uncover the related mechanisms.

## 5. Conclusion

We have built a visually comprehensive nomogram based on TCGA database and incorporated genomic and clinicopathologic factors for the prediction of survival in patients with LSCC. The nomogram is significantly better than TNM stage alone in terms of the predictive value and clinical usability. Importantly, our nomogram presented good discrimination ability in early LSCC and advanced LSCC subgroups.

## Figures and Tables

**Table 1:** Characteristics of study population with the number of missing values (*n* = 109).

		No. (%) or median (IQR)	Missing values (%)
Variable	Category		
Age (years)		62 (38-83)	0 (0)
Sex	Male	90 (82.6)	0 (0)
Smoking history	Yes	46 (42.2)	3 (2.8)
Alcohol history	Yes	69 (63.3)	2 (1.8)
Number of lymph nodes		36 (0-121)	18 (16.5)
Number of positive LNs		1 (0-42)	18 (16.5)
Lymph node ratio		0.18 (0-1)	18 (16.5)
Margin status			15 (13.8)
	Negative	84 (77.1)	
	Positive	10 (9.2)	
Tumor status			6 (5.5)
	Tumor free	73 (67)	
	With tumor	30 (27.5)	
Tumor grade			4 (3.7)
	G1-G2	76 (69.7)	
	G3-G4	29 (26.6)	
Clinical T			4 (3.7)
	T1-T2	19 (17.4)	
	T3-T4	86 (78.9)	
Clinical N			6 (5.5)
	N0	53 (48.6)	
	N1-N3	50 (45.9)	
Clinical stage			4 (3.7)
	I-II	13 (11.9)	
	III-IV	92 (84.4)	
Mutation count		153 (36-861)	2 (1.8)
Fraction genome altered		0.30 (0-0.89)	1 (0.9)

Abbreviations: IQR = interquartile range; LN = lymph node.

**Table 2:** Univariable and multivariable Cox regression analysis for prediction of OS.

Factors	Subgroup	Univariable analysis		Multivariable analysis	
		HR (95% CI)	*P*	HR (95% CI)	*P*
Age		1.00 (0.97-1.04)	0.984	NA	NA
Sex	Female	1			
	Male	0.28 (0.14-0.56)	0.001^∗^	0.45 (0.31-1.07)	0.090
Smoking history	No	1			
	Yes	0.65 (0.35-1.18)	0.156	NA	NA
Alcohol history	No	1			
	Yes	0.77 (0.43-1.39)	0.388	NA	NA
Number of lymph nodes		1.00 (0.98-1.01)	0.558	NA	NA
				
Number of positive LNs		1.00 (0.95-1.04)	0.892	NA	NA
				
Lymph node ratio		1.41 (0.28-7.13)	0.675	NA	NA
Margin status	Negative	1		1	
	Positive	4.68 (2.08-10.51)	0.001^∗^	3.00 (1.47-6.10)	0.003^∗^
Tumor status	Tumor free	1		1	
	With tumor	4.10 (2.23-7.55)	0.001^∗^	3.25 (1.79-5.90)	0.001^∗^
Tumor grade	G1-G2	1			
	G3-G4	0.51 (0.25-1.04)	0.064	NA	NA
Clinical T	T1-T2	1			
	T3-T4	0.72 (0.35-1.50)	0.376	NA	NA
Clinical N	N0	1			
	N1-N3	1.44 (0.80-2.57)	0.222	NA	NA
Clinical stage	I-II	1			
	III-IV	0.86 (0.36-2.03)	0.729	NA	NA
Mutation count		0.99 (0.98-1.01)	0.542	NA	NA
Fraction genome altered		1.44 (0.29-7.18)	0.654	NA	NA
lncRNA signature		1.22 (1.14-1.31)	0.001^∗^	1.21 (1.11-1.31)	0.001^∗^

Abbreviations: HR=hazard ratio; CI=confidence intervals; OS=overall survival; NA=not available. These variables were eliminated in the multivariate Cox regression model, so the HR and *P* values were not available. ^∗^*P* < 0.05.

**Table 3:** Assessing the prognostic performance of the TNM stage, lncRNA signature, and nomogram.

Model	Homogeneity, monotonicity, and discriminatory ability			
	Likelihood ratio (LR) test^∗^	Linear trend *χ*^2^ test^∗∗^	C-index (95% CI)^∗∗∗^	Akaike information criterion (AIC)^∗∗∗∗^
TNM stage	00.5	00.5	0.53 (0.45-0.61)	380.3
lncRNA signature	21.4	46.9	0.78 (0.71-0.85)	355.0
Nomogram	48.0	71.7	0.82 (0.77-0.87)	332.4

^∗^Higher homogeneity likelihood ratio indicates a smaller difference within the staging system, and it means better homogeneity. ^∗∗^Higher discriminatory ability linear trend indicates a higher linear trend between staging systems, and it means a better discriminatory ability and gradient monotonicity. ^∗∗∗^A higher C-index means better discriminatory ability. ^∗∗∗∗^Smaller AIC values indicate better optimistic prognostic stratification.

## Data Availability

The data that support the findings of this study are provided in the supplementary materials and is also made available in TCGA via the Genomics Data Commons (https://gdc.cancer.gov/).

## Abbreviations

LSCC: Laryngeal squamous cell carcinoma
RNA-seq: RNA sequencing
TCGA: The Cancer Genome Atlas
lncRNAs: Long noncoding RNAs
LASSO: Least absolute shrinkage and selection operator
ROC: Receiver operating characteristic
C-index: Concordance index
OS: Overall survival
AUC: Area under curve
DCA: Decision curve analysis
AJCC: American Joint Committee on Cancer
TNM: Tumor node metastasis
LNR: Lymph node ratio
LN: Lymph nodes
GO: Gene ontology
KEGG: Kyoto Encyclopedia of Genes and Genomes
IQRs: Interquartile ranges
SDs: Standard deviations
MI: Multiple imputation
MCMC: Markov Chain Monte Carlo
MF: Molecular function
BP: Biological process
CC: Cellular component
LNM: Lymph node metastasis
GEO: Gene Expression Omnibus
DFS: Disease-free survival
NSCLC: Non-small-cell lung cancer
OSCC: Oral squamous cell carcinoma
HPV: Human papillomavirus.

## Supplementary Materials

Supplementary 1 Supplementary material 1: Based on the primary filter criteria mentioned in Materials and Methods, we obtained a list of 1160 different lncRNAs.

Supplementary 2 Supplementary material 2: Using univariable Cox regression analysis, we identified 31 prognostic-related lncRNAs.

Supplementary 3 Supplementary Figure S1: Kaplan-Meier's survival analysis according to the lncRNA signature stratified by clinicopathological factors. (A) T stage—T1 to T2; (B) T stage—T3 to T4; (C) lymph node status—node negative; (D) lymph node status—node positive; (E) TNM stage—stages I-II, (F) TNM stage—stages III-IV. *P* values were calculated using the log-rank test.

Supplementary 4 Supplementary Figure S2: Calibration curves for the nomogram at different time points. Patients were grouped by octiles of predicted risk. *x*-axis is the nomogram-predicted probability of survival (LSCC). *y*-axis is the observed probability of LSCC (the Kaplan-Meier estimates). Broken line=ideal nomogram; circles=apparent predictive accuracy, calculated by plotting the mean Kaplan-Meier estimate for each octile versus the mean nomogram-predicted probabilities for patients in each octile; *X*'s=bootstrap-corrected estimates; vertical bars=95% CIs.

Supplementary 5 Supplementary Figure S3: Kaplan-Meier curves of three groups based on the score generated by the nomogram. *P* values were calculated using the log-rank test.
