# Supplementary figures and images for: A Genomic-Clinicopathologic Nomogram Predicts Survival for Patients with Laryngeal Squamous Cell Carcinoma
Source: Dis Markers. 2019 Nov 20;2019:5980567. doi: 10.1155/2019/5980567 (PMC6886334; doi:10.1155/2019/5980567)

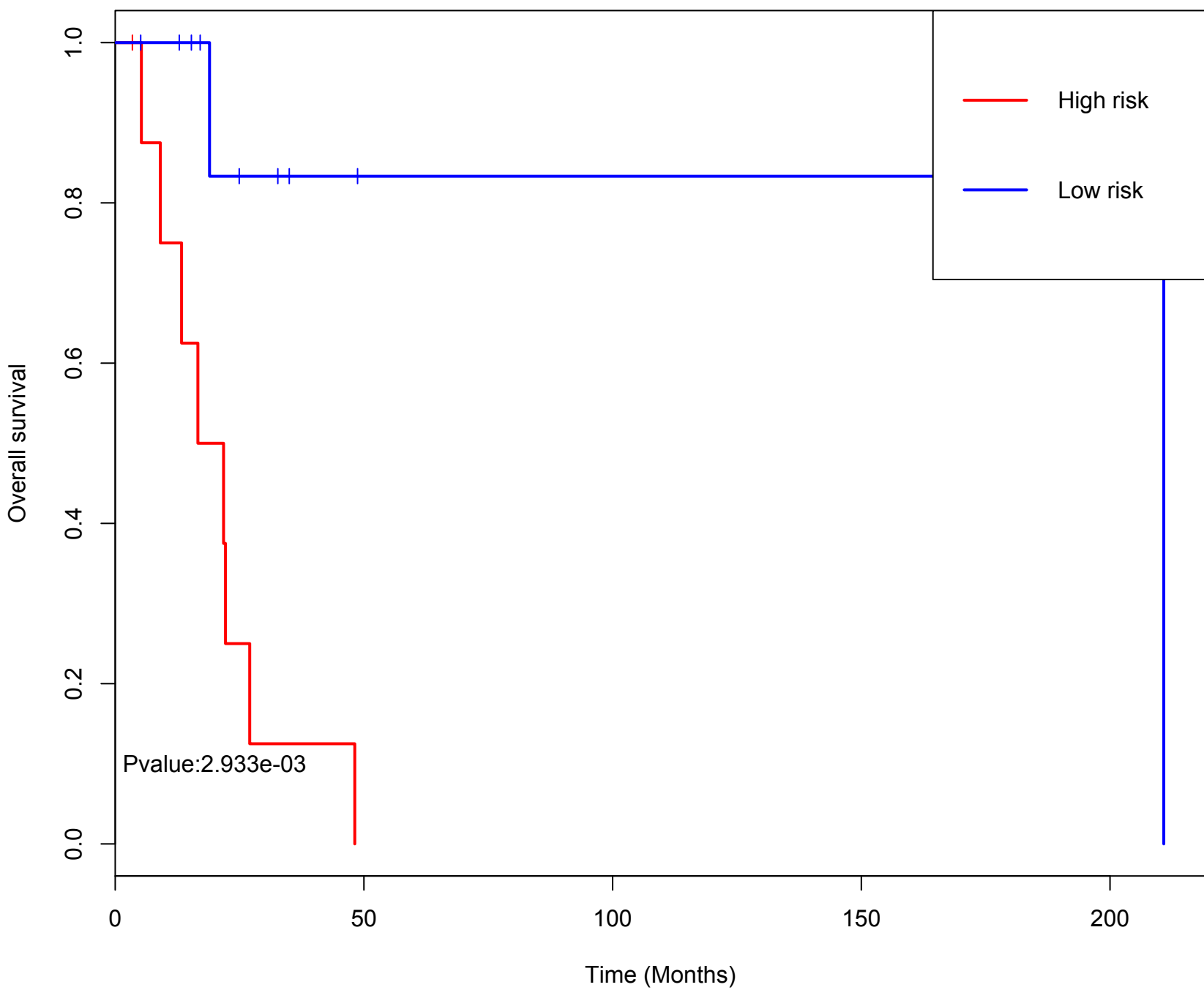

Supplement: Supplementary 3 — Supplementary Figure S1: Kaplan-Meier's survival analysis according to the lncRNA signature stratified by clinicopathological factors. (A) T stage—T1 to T2; (B) T stage—T3 to T4; (C) lymph node status—node negative; (D) lymph node status—node positive; (E) TNM stage—stages I-II, (F) TNM stage—stages III-IV. P values were calculated using the log-rank test. [file 5980567.f3.zip › Supplementary FigureS1A.pdf]

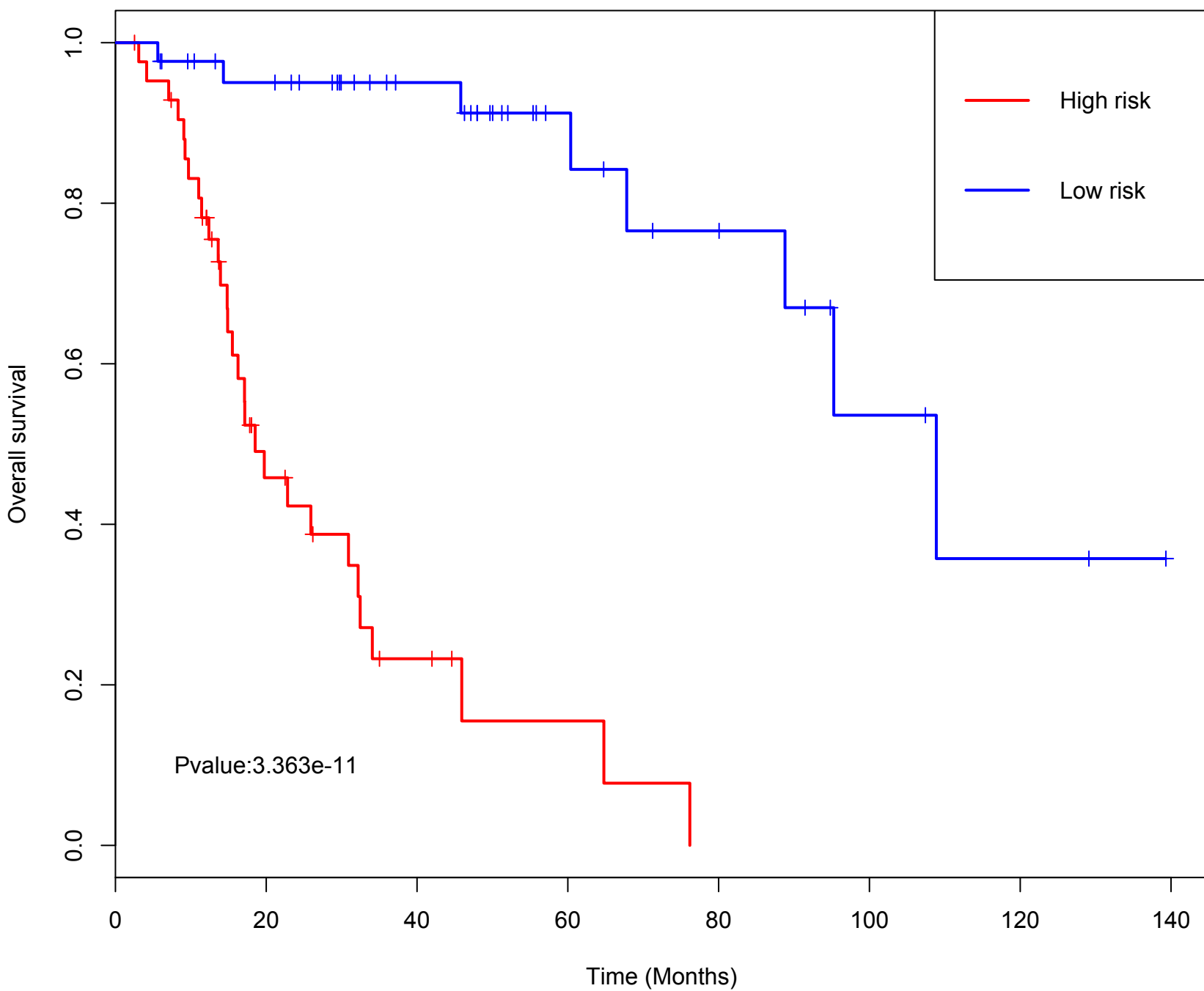

Supplement: Supplementary 3 — Supplementary Figure S1: Kaplan-Meier's survival analysis according to the lncRNA signature stratified by clinicopathological factors. (A) T stage—T1 to T2; (B) T stage—T3 to T4; (C) lymph node status—node negative; (D) lymph node status—node positive; (E) TNM stage—stages I-II, (F) TNM stage—stages III-IV. P values were calculated using the log-rank test. [file 5980567.f3.zip › Supplementary FigureS1B.pdf]

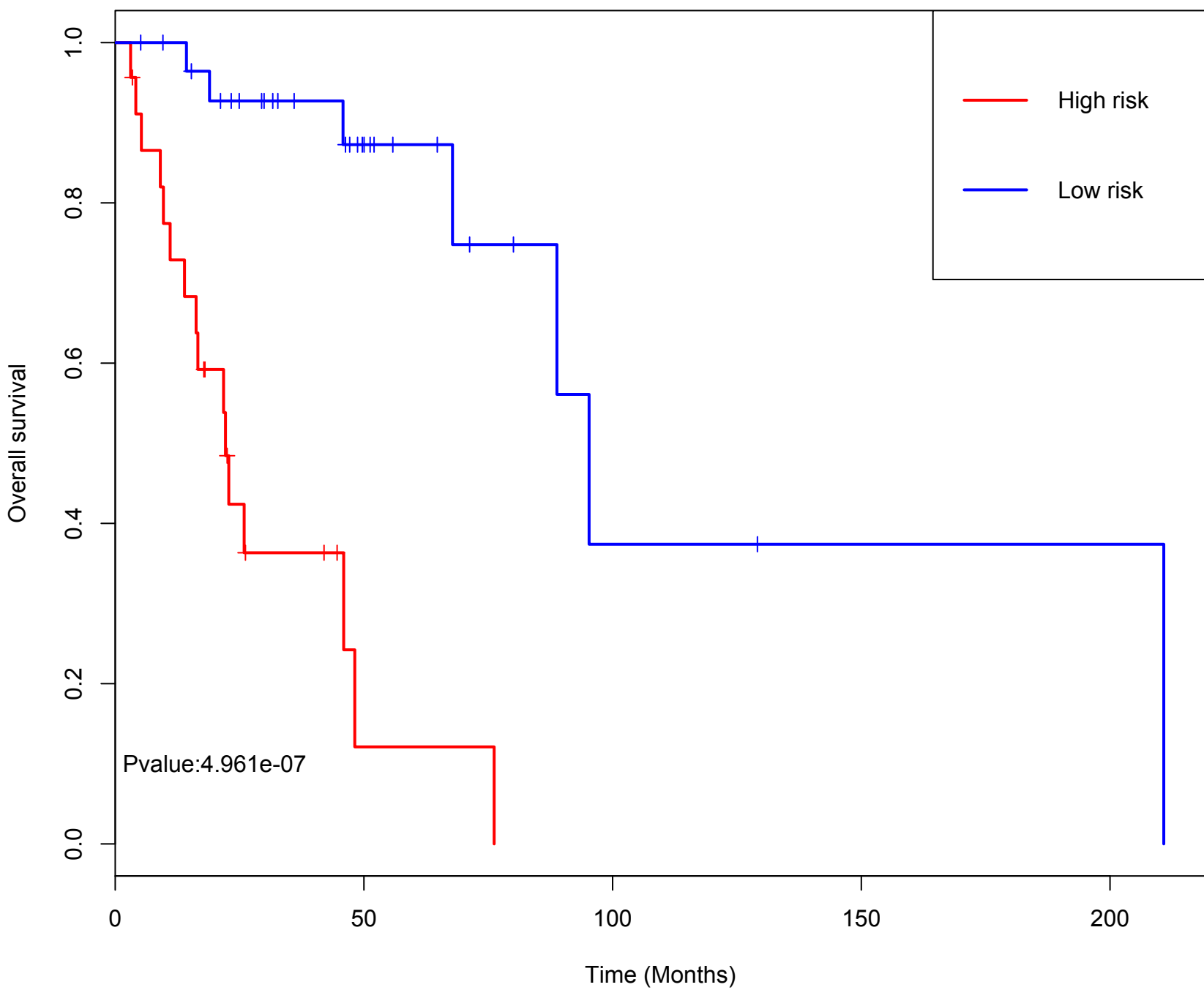

Supplement: Supplementary 3 — Supplementary Figure S1: Kaplan-Meier's survival analysis according to the lncRNA signature stratified by clinicopathological factors. (A) T stage—T1 to T2; (B) T stage—T3 to T4; (C) lymph node status—node negative; (D) lymph node status—node positive; (E) TNM stage—stages I-II, (F) TNM stage—stages III-IV. P values were calculated using the log-rank test. [file 5980567.f3.zip › Supplementary FigureS1C.pdf]

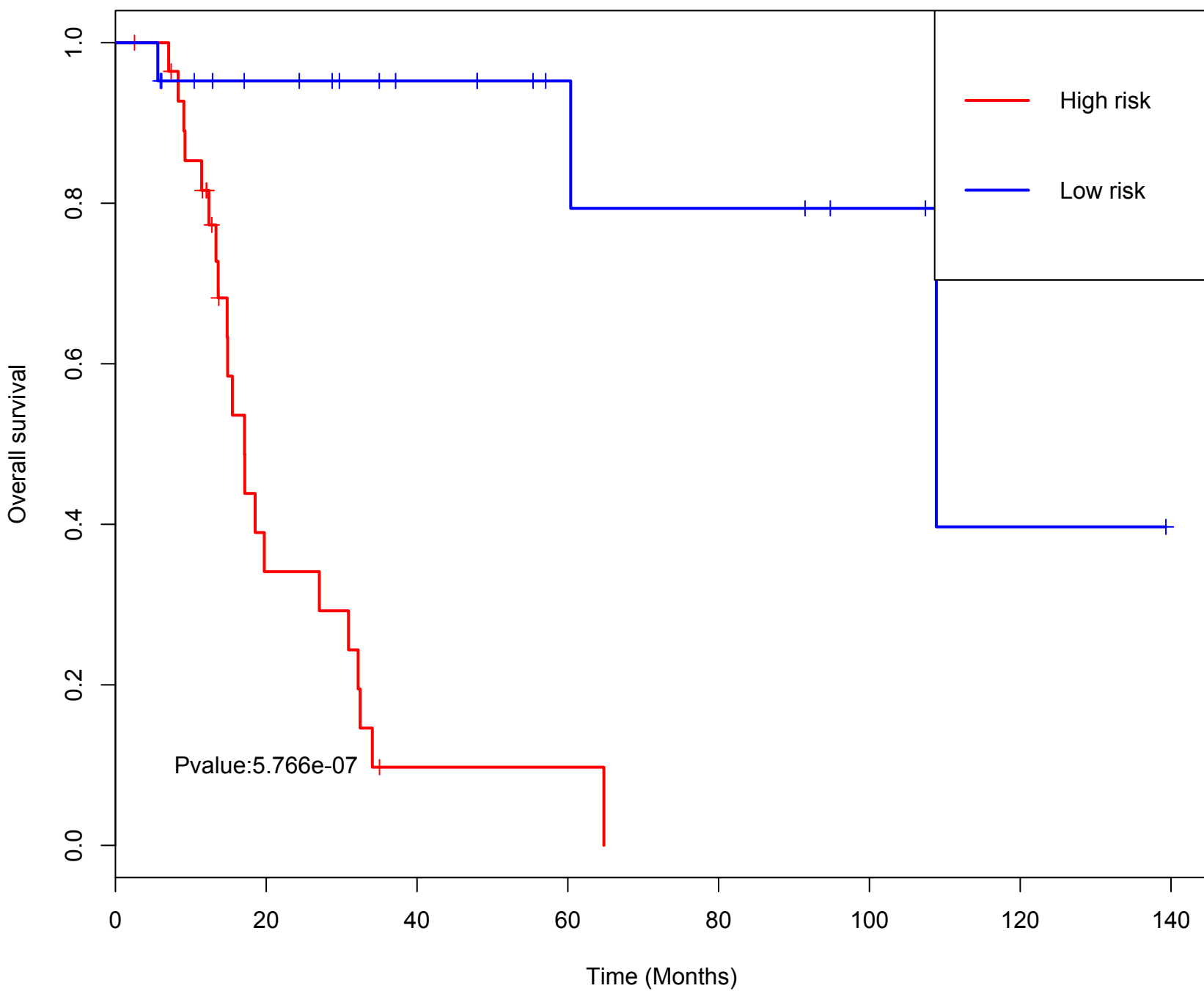

Supplement: Supplementary 3 — Supplementary Figure S1: Kaplan-Meier's survival analysis according to the lncRNA signature stratified by clinicopathological factors. (A) T stage—T1 to T2; (B) T stage—T3 to T4; (C) lymph node status—node negative; (D) lymph node status—node positive; (E) TNM stage—stages I-II, (F) TNM stage—stages III-IV. P values were calculated using the log-rank test. [file 5980567.f3.zip › Supplementary FigureS1D.pdf]

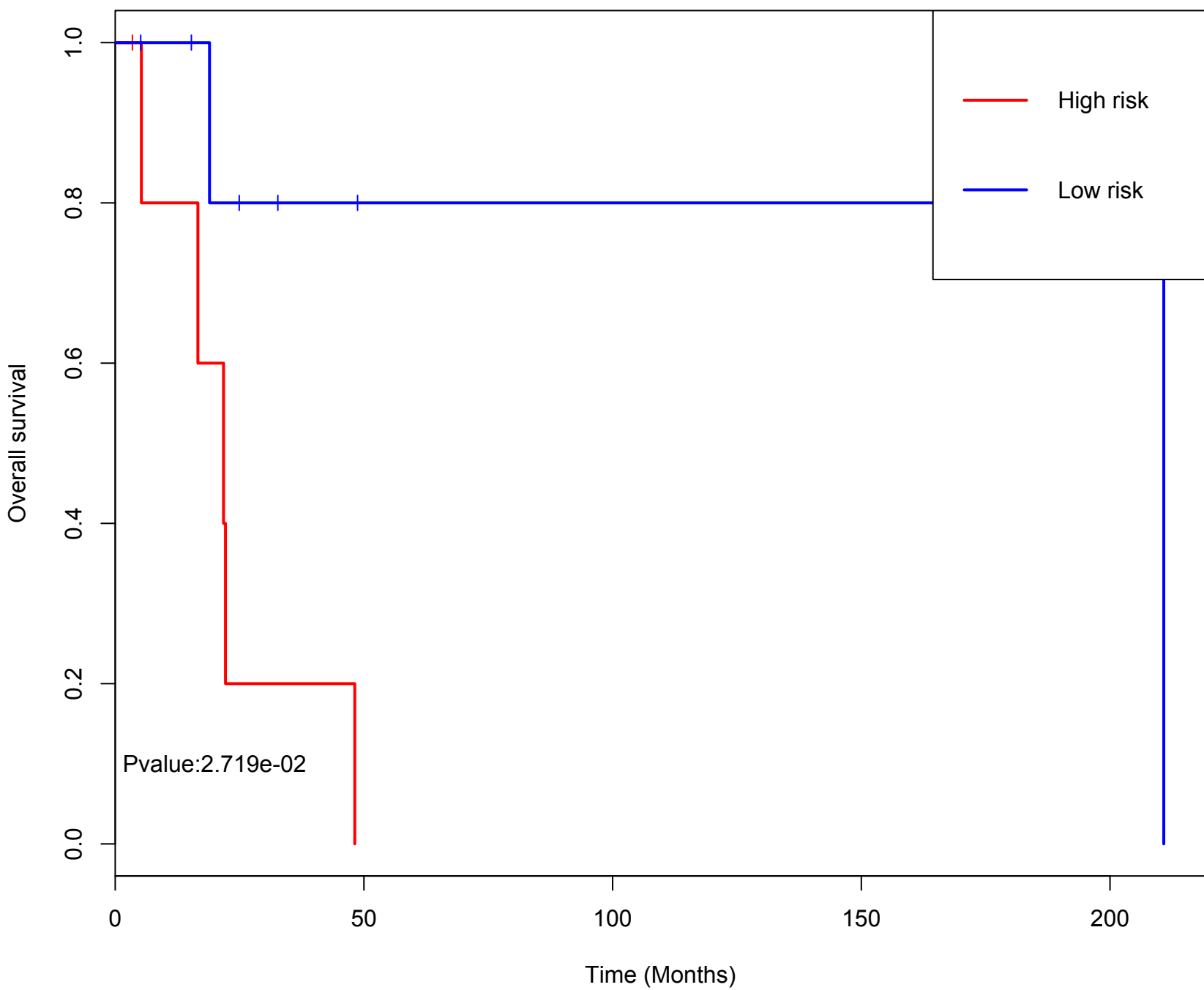

Supplement: Supplementary 3 — Supplementary Figure S1: Kaplan-Meier's survival analysis according to the lncRNA signature stratified by clinicopathological factors. (A) T stage—T1 to T2; (B) T stage—T3 to T4; (C) lymph node status—node negative; (D) lymph node status—node positive; (E) TNM stage—stages I-II, (F) TNM stage—stages III-IV. P values were calculated using the log-rank test. [file 5980567.f3.zip › Supplementary FigureS1E.pdf]

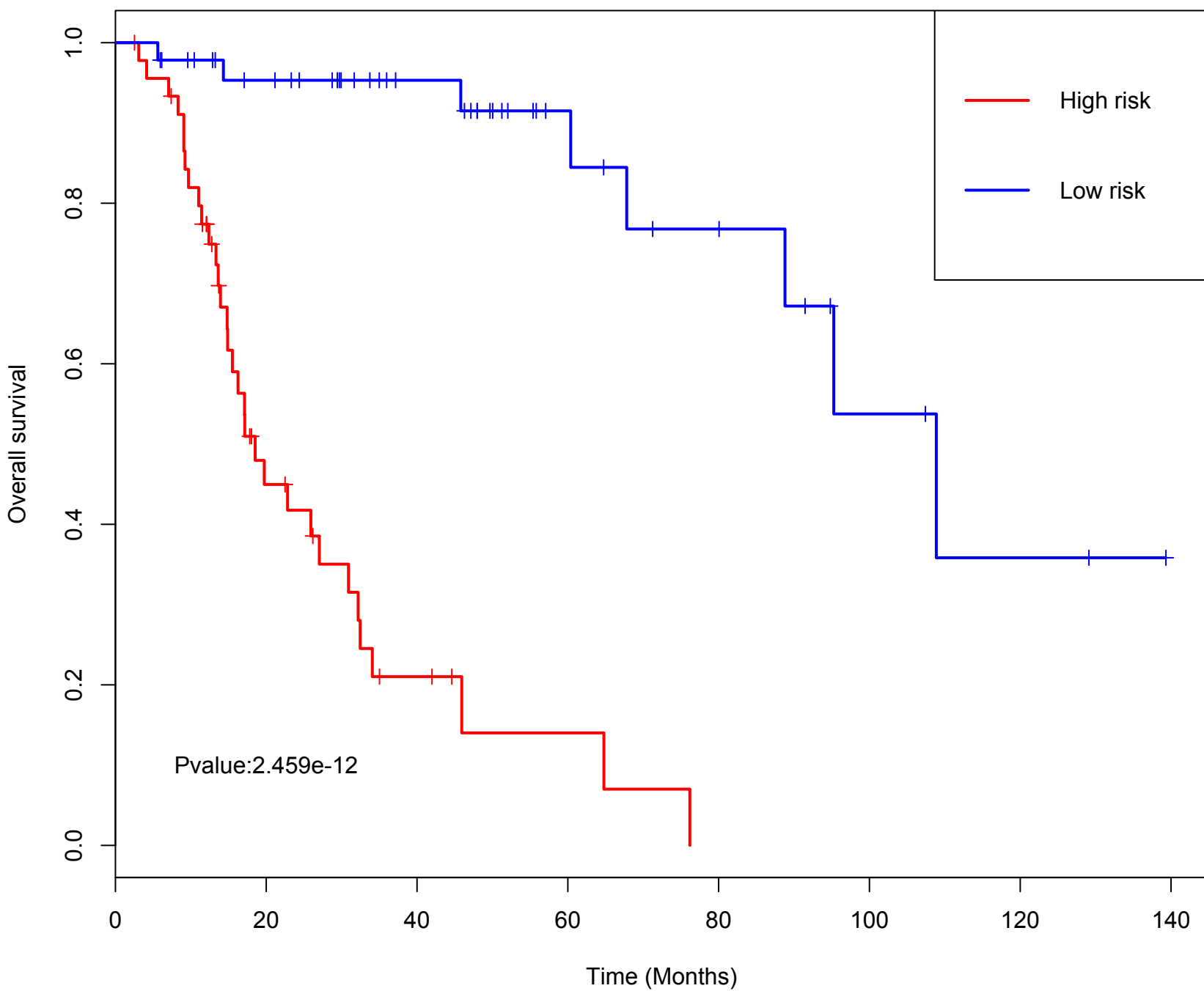

Supplement: Supplementary 3 — Supplementary Figure S1: Kaplan-Meier's survival analysis according to the lncRNA signature stratified by clinicopathological factors. (A) T stage—T1 to T2; (B) T stage—T3 to T4; (C) lymph node status—node negative; (D) lymph node status—node positive; (E) TNM stage—stages I-II, (F) TNM stage—stages III-IV. P values were calculated using the log-rank test. [file 5980567.f3.zip › Supplementary FigureS1F.pdf]

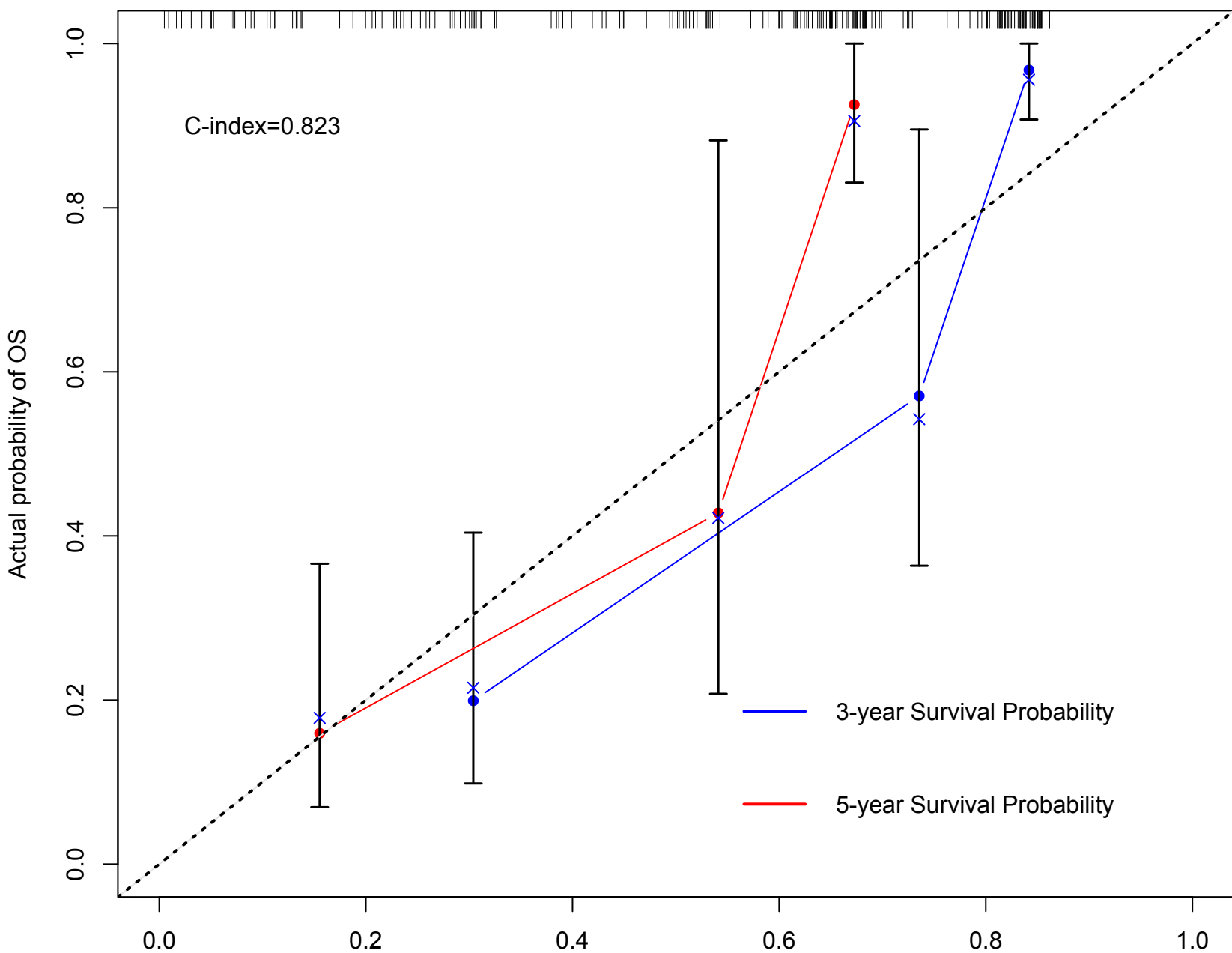

n=109 d=49 p=3, 30 subjects per group  
Gray: ideal

X - resampling optimism added, B=998  
Based on observed-predicted

Supplement: Supplementary 4 — Supplementary Figure S2: Calibration curves for the nomogram at different time points. Patients were grouped by octiles of predicted risk. x-axis is the nomogram-predicted probability of survival (LSCC). y-axis is the observed probability of LSCC (the Kaplan-Meier estimates). Broken line=ideal nomogram; circles=apparent predictive accuracy, calculated by plotting the mean Kaplan-Meier estimate for each octile versus the mean nomogram-predicted probabilities for patients in each octile; X's=bootstrap-corrected estimates; vertical bars=95% CIs. [file 5980567.f4.pdf]

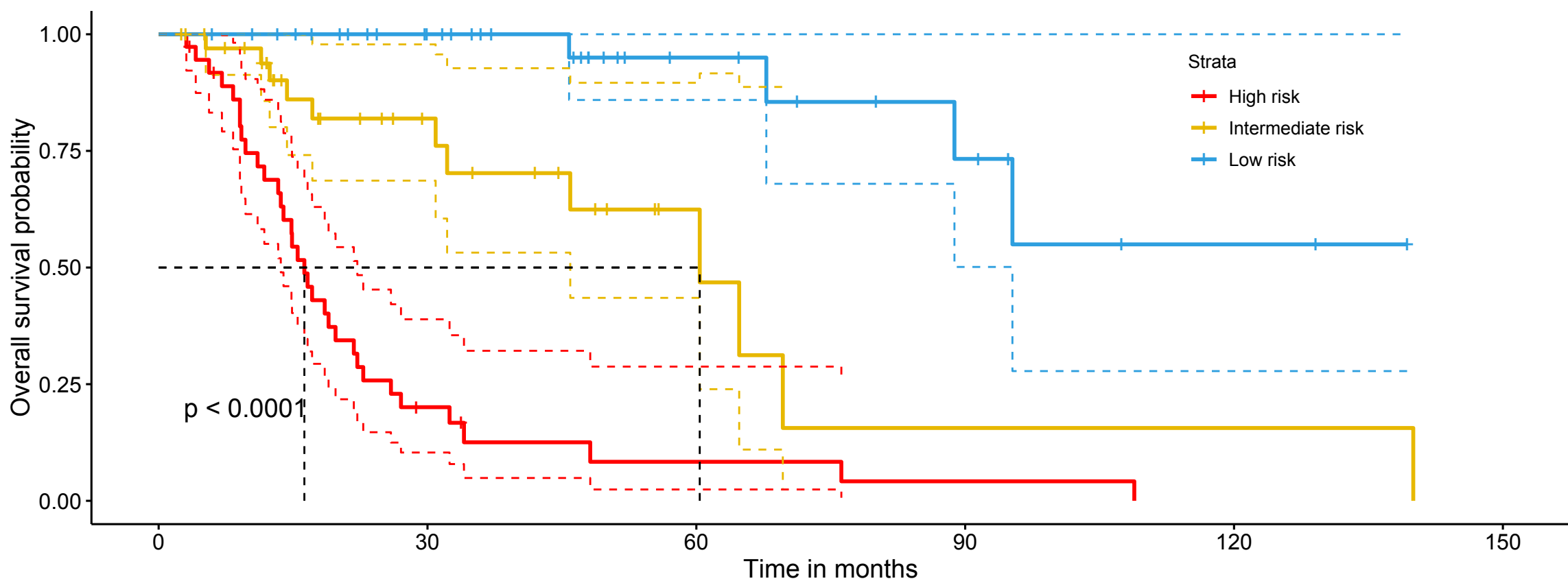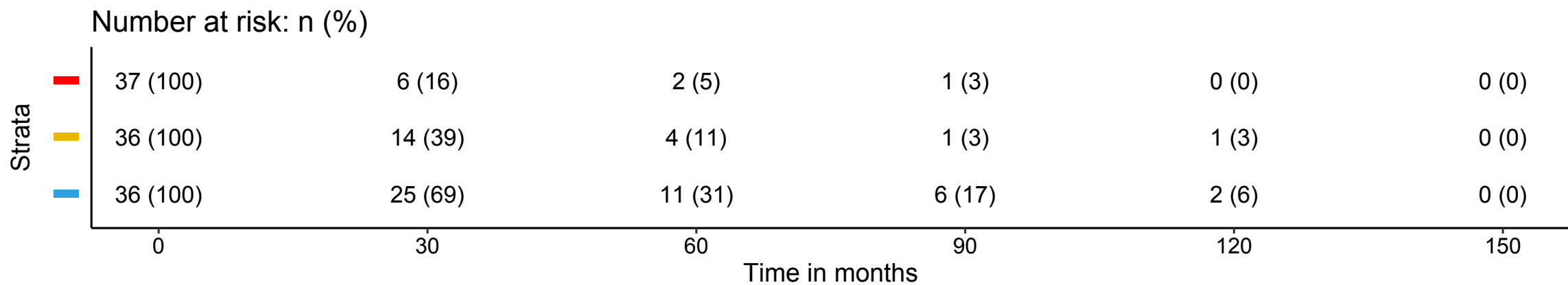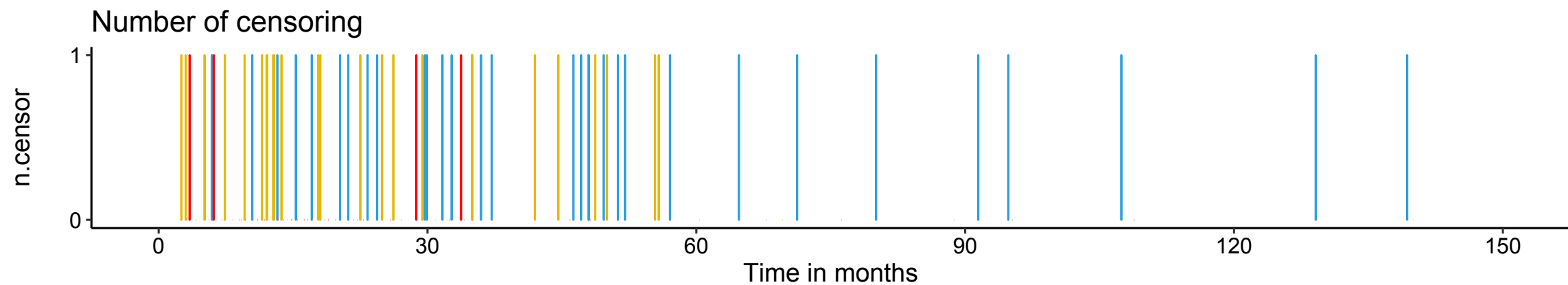

Supplement: Supplementary 5 — Supplementary Figure S3: Kaplan-Meier curves of three groups based on the score generated by the nomogram. P values were calculated using the log-rank test. [file 5980567.f5.pdf]
